# Successful Treatment of Hip Osteoarthritis With Radiofrequency Ablation: A Report of Two Cases

**DOI:** 10.7759/cureus.80122

**Published:** 2025-03-06

**Authors:** Toshio Okada, Toru Goyagi, Kiyoshige Ohseto

**Affiliations:** 1 Department of Anesthesiology, Tokyo Medical University Hospital, Tokyo, JPN

**Keywords:** chronic pain management, hip joint pain, nerve block, osteoarthritis of the hip, radiofrequency ablation (rfa)

## Abstract

Osteoarthritis (OA) is the most common cause of activity limitation in adults. In two patients with chronic pain due to hip OA who had inadequate pain relief or had severe recalcitrant pain, fluoroscopy-guided radiofrequency ablation (RFA) of the hip joint nerve branches was performed, and analgesic effects were obtained. RFA of the hip joint nerve branches is usually performed by taking into consideration the localization of the femoral nerve and the obturator nerve innervating the anterior aspect of the hip joint, and the superior gluteal nerve and sciatic nerve innervating the posterior aspect of the hip joint. In these cases, RFA can be performed more easily by using a direct needle approach and can be more specific about tender points. These two cases suggest the usefulness of hip nerve branches RFA for patients with chronic pain due to hip OA who may not be candidates for total hip arthroplasty.

## Introduction

Osteoarthritis (OA) is the most common cause of activity limitation in elderly people, affecting an estimated 240 million individuals worldwide. Conservative treatments, intra-articular steroid injections, and duloxetine have been proven effective [1]. The lifetime risk for people up to 85 is estimated at 25% for symptomatic hip OA [2]. The lifetime risk of having a total hip replacement in end-stage OA is also almost 10% [3]. Patients with advanced symptoms and structural damage are candidates for total hip arthroplasty (THA), but those who are unable or unwilling to undergo surgery due to medical complications may benefit from interventional treatment with nerve blocks. Approximately 7% to 23% of patients have residual pain after hip replacement [4], and nerve blocks may be necessary. In this study of two patients with chronic pain due to hip OA who had difficulty controlling their pain, fluoroscopic-guided radiofrequency ablation (RFA) of the hip joint nerve branch was performed, and analgesia was obtained. RFA could be performed more easily by directly approaching the tender point of the hip joint with a needle, rather than using the conventional method that takes into account the localization of the hip joint nerve branch.

## Case presentation

Case 1

**Figure 1:**
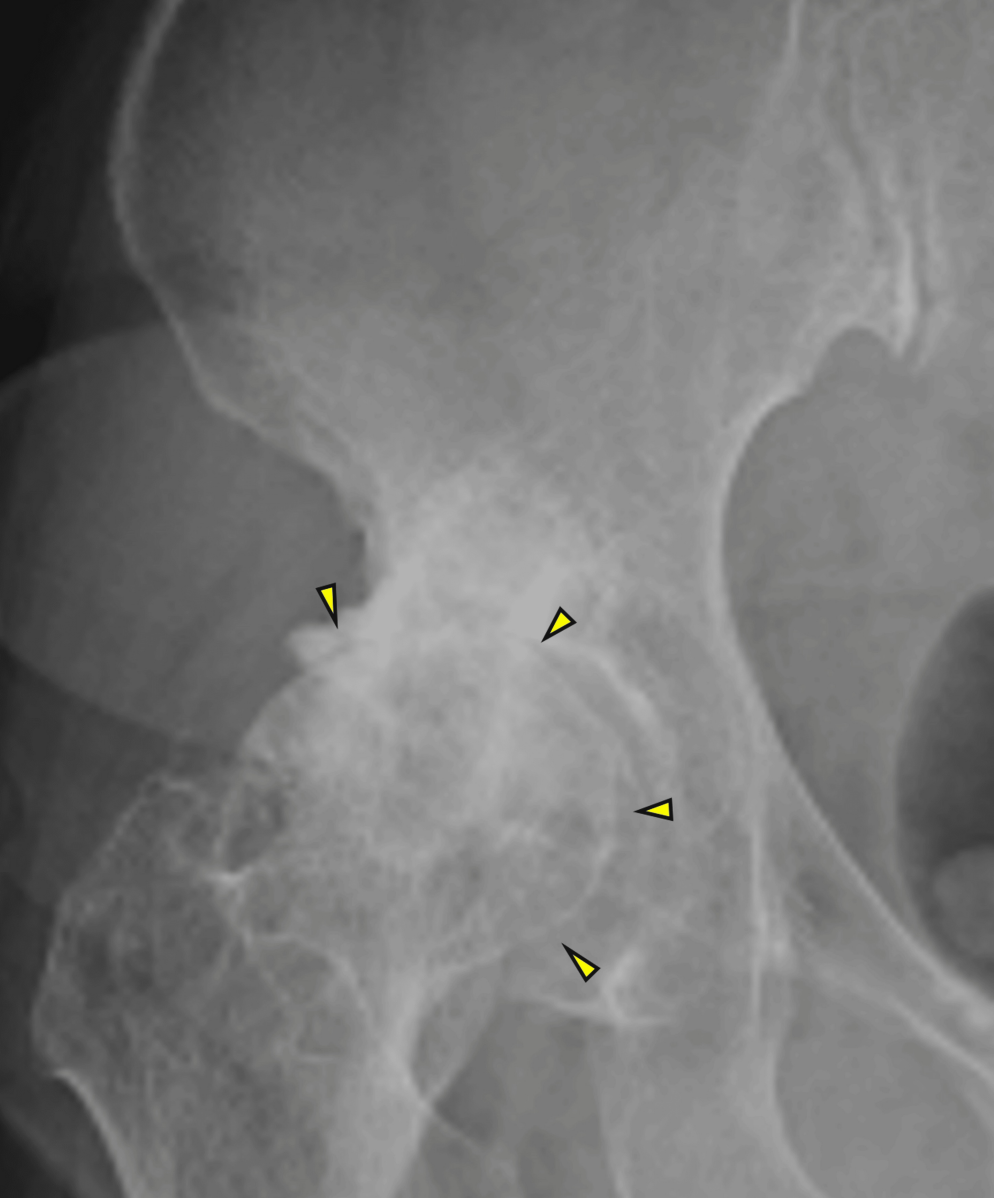
X-rays of the right hip joint in patient 1 Arrows indicate the narrowing of the right hip joint space.

**Figure 2:**
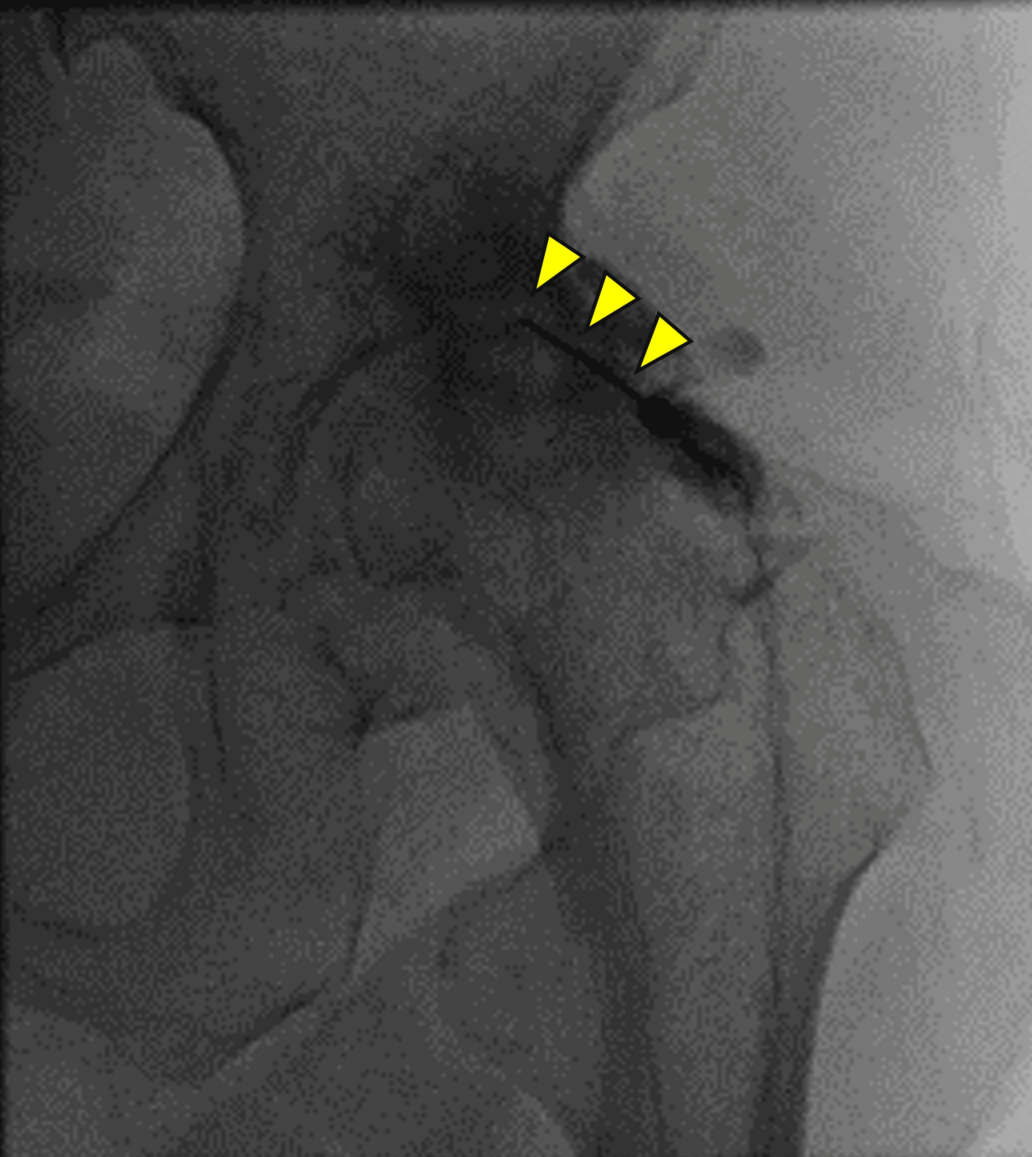
Fluoroscopic image of radiofrequency ablation in patient 1 A needle (arrowheads) is inserted into a tender point on the right hip joint, and RFA is performed at 80°C for 90 seconds.

An 80-year-old woman with right hip pain with a numerical rating scale (NRS) score of 8/10 and numbness in the right lower extremity for the past year presented to our hospital. The patient had a history of rheumatoid arthritis and was taking methotrexate 4 mg/week. X-rays showed narrowing of the right hip joint space (Figure 1) and lumbar spondylolisthesis at L4/5. Since the patient did not wish to have surgery, the plan was for interventional treatment. After performing a lumbar plexus block and sciatic nerve block, the patient remained in pain (NRS score 5/10), so the right hip nerve RFA was performed. The patient was placed in the supine position, the needle was inserted targeting the tender point on the right hip, and after confirming reproducible pain by stimulating at 0.3 mA, RFA was performed at 80°C for 90 seconds (Figure 2). Prior to performing RFA, 1 ml of 1% lidocaine was injected to tolerate the pain caused by RFA stimulation. The right hip pain improved to an NRS score of 2/10, and the pain has been controlled with oral medication alone for 18 months.

Case 2

**Figure 3:**
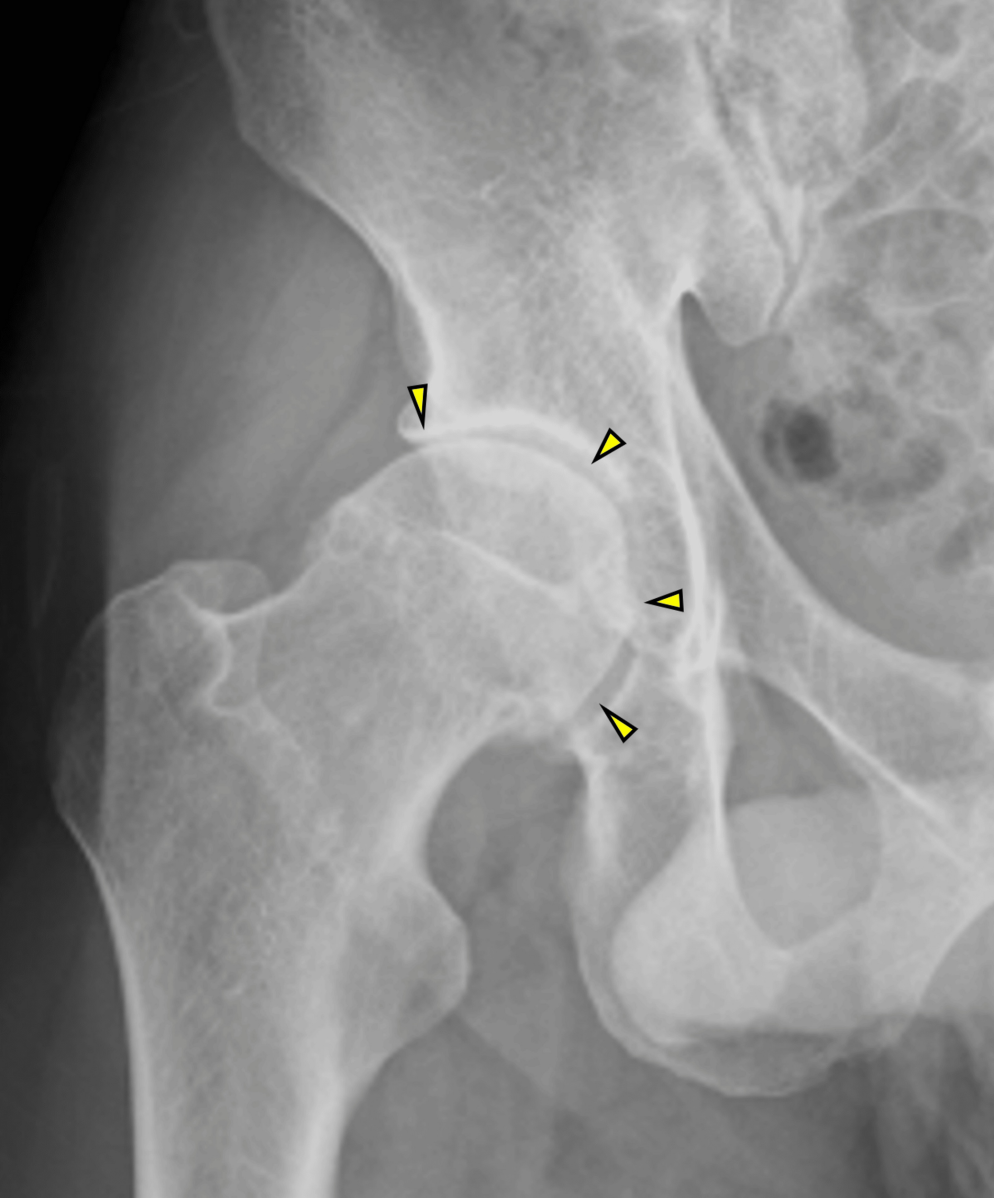
X-rays of the right hip joint in patient 2 Arrows indicate the narrowing of the right hip joint space.

**Figure 4:**
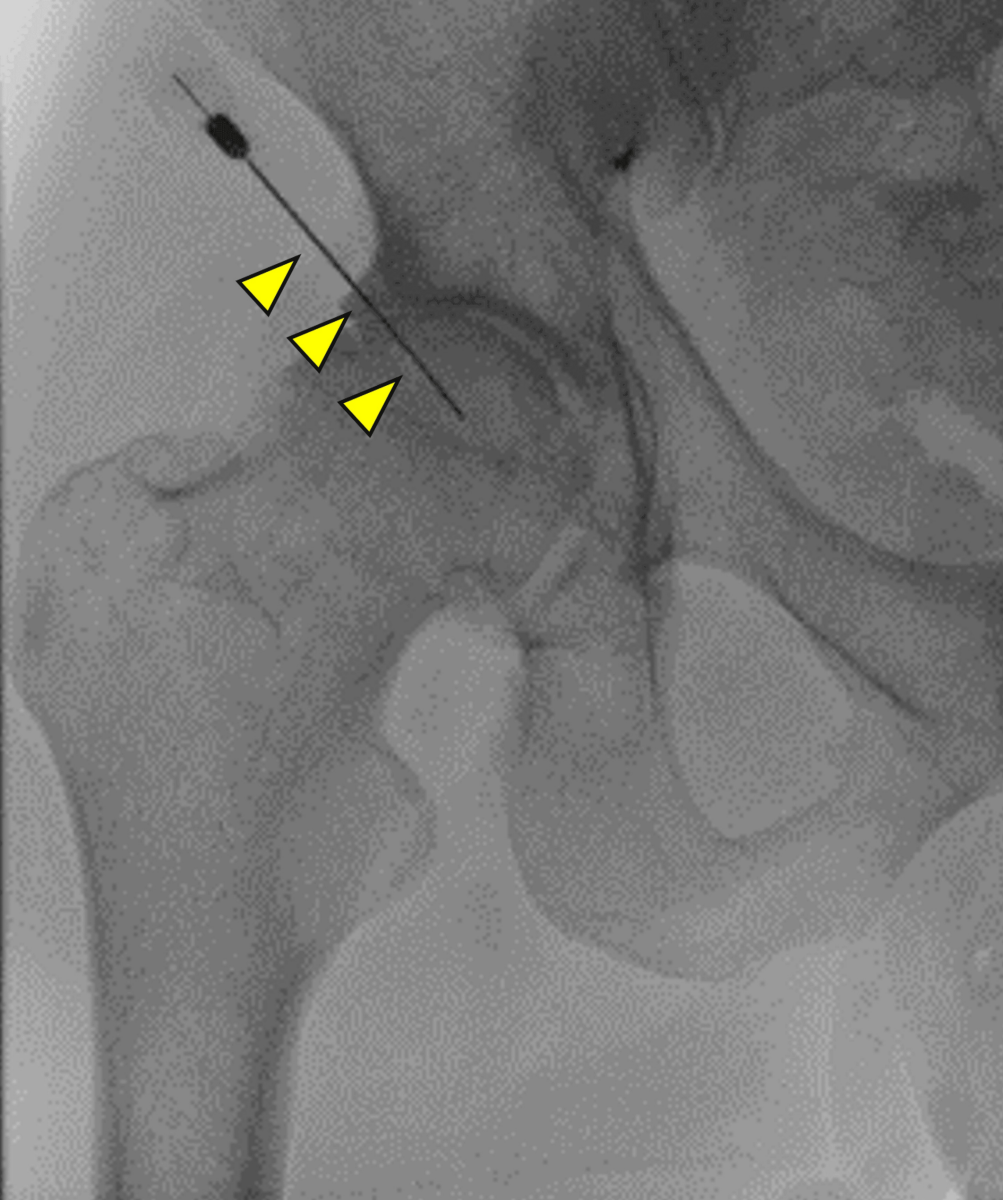
Fluoroscopic image of radiofrequency ablation in patient 2 A needle (arrowheads) is inserted into a tender point of the right hip joint, and RFA is performed at 80°C for 90 seconds.

A 50-year-old man with an NRS score of 8/10 for right hip pain and numbness in the right lower extremity for the past five years presented to our hospital. The patient had a history of surgery for lumbar spondylolisthesis. X-rays showed a narrowing of the right hip joint space (Figure 3). Symptoms did not improve with oral administration of 2 tramadol hydrochloride/acetaminophen tablets and pregabalin 150 mg/day. Since the patient did not wish to have surgery, the plan was for interventional treatment. Lumbar plexus block and sciatic nerve block were performed, which improved the symptoms temporarily. However, the pain of NRS score 8/10 flared up, so right hip nerve RFA was performed. The patient was placed in the supine position, and the needle was inserted targeting the tender point of the right hip joint while confirming the absence of blood vessels in the entry path by echo, and RFA was performed at 80°C for 90 seconds (Figure 4). Prior to performing RFA, 1 ml of 1% lidocaine was injected to tolerate the pain caused by RFA stimulation. The pain in the right hip joint improved to an NRS score of 0/10 after RFA, and the pain has been controlled with oral medication alone until now, 12 months later.

 In both cases, no complications such as paralysis of the lower extremities occurred after RFA.

## Discussion

In a review of 14 articles on RFA for the hip nerve branch, all showed high analgesic efficacy up to 36 months after the procedure without serious complications [5]. Pulsed radiofrequency (PRF) also demonstrated good analgesia and increased quality of life in the Oxford Hip Score (OHS) up to six months after the procedure, but the NRS score and OHS were 5.8/10 and 27.4, respectively, at 12 months, and the long-term results are unknown [6]. Although RFA is associated with potential risks of neuritis and neuroma formation [7], it may be an option for patients who cannot be treated with the relatively safe PRF.

The sensory nerves of the hip joint are innervated by the femoral nerve and the obturator nerve on the anterior aspect and by the superior gluteal nerve and sciatic nerves on the posterior aspect [8,9]. Case 1 had tender points on the posterior aspect of the hip joint, and a branch of the sciatic nerve was involved, whereas Case 2 had tender points on the anterior aspect of the hip joint, and a branch of the femoral nerve was involved. A cadaver study of the innervation of the anterior aspect of the hip capsule showed that the femoral and obturator nerves all supply nerve endings to the anterior aspect of the hip joint, and in about half of the cases, the accessory obturator nerve was also reported to supply a branch [10]. As landmarks for each nerve, the femoral nerve was reported to be located between the anterior inferior iliac spine and the iliopubic ramus, the obturator nerve was reported to be located medial to the acetabulum (radiographic “tear drop”), and the accessory obturator nerve was reported to be located at the iliopubic ramus [10]. Recently, an ultrasound-guided block known as the pericapsular nerve group block, targeting landmarks between the anterior inferior iliac spine and the iliopubic ramus [11], has shown potential for perioperative pain management in THA by blocking the femoral and accessory obturator nerves. There have been no anatomical studies describing in detail the nerve landmarks on the posterior surface of the hip capsule. Thus, the hip joint nerve branches of each nerve can be inferred to some extent from the landmarks and are the target for RFA.

Conventional hip joint nerve branch RFA is performed taking into account the locations of the hip joint nerve branches of the femoral nerve, the obturator nerve, the superior gluteal nerve, and the sciatic nerve [8,9], but in actual practice, it is sometimes difficult to obtain radiating pain even when joint nerve branches are explored with electrical stimulation, and the effect is sometimes insufficient. The reasons for this may include the existence of high and low branches of both the femoral nerve and the obturator nerve, individual differences in the supply of peripheral branches to the hip joint, and the presence of the accessory obturator nerve.

Since the method used in the present study targets the needle tip at the tender point on the capsule, the technique is simpler than the aforementioned method [8,9], and it may be possible to puncture at the tender point as in intervertebral joint nerve branch RFA. However, it is important to confirm important nerves and blood vessels in the needle path by palpation and ultrasonography.

The anatomy of the location of the articular branches on the acetabulum has not been fully elucidated. The presence of sensory nerve endings (Vater-Pacini corpuscle, Golgi-Mazzoni corpuscle, Ruffini corpuscle, and Krause corpuscle) on the articular side of the labrum has been reported [12], and they may serve as nociceptors for hip pain and be a target for RFA. Further case series are needed to confirm the long-term efficacy of RFA using this technique.

## Conclusions

In recent years, some patients with chronic hip pain have been unable to undergo hip replacement surgery due to multiple comorbidities, and these patients often fail to improve with drug therapy. The present two cases suggest that hip nerve branch RFA may be useful in avoiding surgery and managing pain in patients with chronic pain due to hip OA, which may be an indication for hip replacement surgery.
